# The clinical prediction factors for non-culprit lesion progression in patients with acute ST elevation myocardial infarction after primary percutaneous coronary intervention

**DOI:** 10.1186/s12872-022-02974-2

**Published:** 2022-12-06

**Authors:** Jian Wang, Cheng-ying Yan, Wu Wang, Tian-zhen Wang

**Affiliations:** 1 Department of Cardiology, Beijing Geriatric Hospital, No. 118 Wenquan Road, Haidian District, Beijing, 100095 China; 2 Department of Cardiology, Xining First People’s Hospital, Xining, 810001 Qinghai China; 3 RDFZ Chaoyang Branch School, Beijing, 100028 China

**Keywords:** ST-elevation myocardial infarction, Fasting blood glucose, Non-culprit lesion progression

## Abstract

**Background:**

To investigate the relationship between the clinical features and progression of non-culprit lesions in patients with ST-elevation myocardial infarction (STEMI) after primary percutaneous coronary intervention (PPCI).

**Methods:**

A total of 480 patients (57.1 ± 9.2 y) with STEMI who underwent PPCI between January 2016 and December 2017 in Beijing Anzhen Hospital were enrolled in this study. All patients underwent PPCI as a treatment for culprit lesions. Clinical and angiographic follow-up were performed for 12 months. All patients were divided into a non-culprit lesions (NCL) progression group (205 cases) and a control group (275 cases) based on angiographic follow-up outcomes at 12 months. The clinical and angiographic features were analyzed.

**Results:**

Body mass index (BMI), serum creatinine (Scr), fasting blood glucose (FBG), glycated serum albumin, glycated hemoglobin and homocysteine levels in the NCL progression group were significantly higher than those in the control group (*P* < 0.05). A logistic regression analysis showed that FBG (odds ratio 1.274, 95% confidence interval 1.077–1.505, *P* = 0.005) and Scr (odds ratio 1.020, 95% confidence interval 1.002–1.038, *P* = 0.027) were independent predictors of NCL progression. A partial correlation analysis showed that FBG was positively correlated with NCL progression (*r* = 0.231, *P* = 0.001). A receiver operating characteristic curve showed that the boundary point of FBG to predict NCL progression was 5.715 mmol/L, and the sensitivity was 74.4% and the specificity was 46.4%.

**Conclusion:**

FBG is a valuable predictor for NCL progression in patients with STEMI after PPCI.

## Background

Primary percutaneous coronary intervention (PPCI) can salvage dying myocardium, reduce cardiovascular events, and improve prognosis in patients with ST-elevation myocardial infarction (STEMI). However, recent clinical studies have shown that non-culprit lesions (NCLs) may progress after PPCI and the progression of NCLs could be the most significant factor affecting prognosis after PPCI [1, 2]. However, the clinical correlation factors for NCL progression are not clear. In the current study, we investigate the relationship between the clinical features and the progression of NCL in patients with STEMI after PPCI.

## Methods

All participants or their family members were informed about the potential publication of their identities and images, and all of them completed consent forms. All procedures and protocols were approved by the ethics committee of Beijing Anzhen Hospital, Capital Medical University, and the experiments were conducted in accordance with the Declaration of Helsinki (1975 and subsequent revisions).

Between January 2016 and December 2017, 480 patients (400 men and 80 women) with acute STEMI who underwent PPCI treatment in Beijing Anzhen Hospital were enrolled in this retrospective study. Clinical and angiographic follow-up was performed in all patients for 12 months. The inclusion criteria were as follows. (1) Acute myocardial infarction lasting for < 12 h and only one NCL found in the setting of STEMI. Acute myocardial infarction was defined as follows: evidence of ischemic chest pain lasting for > 30 min, and new ST segment elevation of ≥ 2 mm in two or more contiguous electrocardiographic leads; a de novo lesion; single vessel treatment in a native vessel ≥ 2.5 mm in diameter and occluded, thrombus containing; thrombolysis in myocardial infarction (TIMI) flow grade of 0 to 2 in the culprit artery, and the grade of stenosis of NCLs was < 70%. (2) There was no contraindication for anticoagulation and antiplatelet therapy.

The main exclusion criteria included the following: previous percutaneous coronary intervention (PCI) in an infarction-related artery (IRA) (*n* = 7), Killip class ≥ 3 (*n* = 8), left or right bundle branch block (*n* = 10), IRA with excessive proximal tortuosity or severe calcification (*n* = 13), left ventricular ejection fraction < 35% (*n* = 14,), lack of clinical and angiographic follow-up (*n* = 25), in-hospital death after PPCI (*n* = 10,included 6 patients with cardiac shock ), myocardial infarction within 2 w of PPCI to exclude potential subacute stent thrombosis of the intervened arterial segment (*n* = 8), and repeated PCI of culprit coronary lesions for restenosis or progression (*n* = 41).

Coronary angiography was performed using the Judkins method, and coronary artery lesion classification was based on the American College of Cardiology/American Heart Association guidelines [3]. Thrombus aspiration catheters (DIVER CE, Invatec, Brescia, Italy) were used for thrombotic burden lesions. Stents were implanted using a routine method, and the procedure succeeded with residual stenosis < 20%, TIMI flow grade of 3 and no acute complications (death, myocardial infarction, emergency coronary artery bypass grafting (CABG)), and no major adverse cardiac events (cardiac death, myocardial infarction, target vessel revascularization). Clinical and angiography follow-up was performed for 12 months.

The culprit coronary lesions were clearly identified through a combination of electrocardiography and coronary angiography. NCLs were defined as those with a diameter of stenosis < 70%. All patients underwent PPCI for the culprit lesions.

Quantitative coronary angiography was performed during the first angiography. Follow-up angiography was performed by two independent investigators who were blinded to the results. We categorized the lesions in accordance with the American College of Cardiology/American Heart Association. Classification was performed on the basis of the morphological characteristics of lesions that cause significant stenosis of the coronary arteries [3]. These include two categories of simple lesions (A or B1 lesions) and complex lesions (B2 or C).

The collected data included demographic information, medical history, coronary artery disease risk factor status, detailed coronary angiographic information, biomarkers associated with coronary atherosclerosis at the time of baseline PCI, and coronary angiographic information at the time of angiographic follow-up.

All clinical, laboratory, and coronary angiographic data were evaluated by two independent investigators who were not involved in the angiographic procedures.

Definition of NCL progression [3]: (1) The stenosis degree of the NCL was ≥ 50% at the time of baseline PCI, and the degree of NCL progression was ≥ 10% at the time of angiographic follow-up. (2) The stenosis degree of the NCL was < 50% at the time of baseline PCI, and the degree of NCL progression was ≥ 30% at the time of angiographic follow-up. (3) The degree of NCL progression ≥ 30%, while there were no NCLs at the time of baseline PCI. (4) NCL progression to total occlusion.

Hypertension was defined as systolic blood pressure > 140 mmHg (1 mmHg = 0.133 kPa)/and/or diastolic blood pressure > 90 mmHg, or patients were taking antihypertensive drugs, in accordance with the 2010 Chinese Hypertension Prevention Guide revised edition [4].

Diabetes was defined as the typical symptoms of diabetes (drinking more, polyphagia, polyuria, weight loss) and fasting plasma glucose > 7.0mmol/L or blood sugar > 11.1mmol/L 2 h after an oral glucose tolerance test, in accordance with the China Guideline for the Prevention of Type 2 Diabetes (2017 Edition) [5].

SPSS20.0 software was used for all statistical analyses. Count data are expressed as cases and percentages, and the χ^2^ test was used for analysis. Numerical data are expressed as mean ± SD and were compared using the Student’s t test. Non-normally distributed numerical data are expressed as the median and 25th–75th interquartile range and were compared using a rank-sum test. A partial correlation analysis was used to evaluate the correlations between fasting blood glucose (FBG), and progression of NCL. Binary logistic regression analysis was performed to examine independent risk factors for the progression of NCL. Receiver-operating characteristic (ROC) analysis and a calculation of sensitivity and specificity were performed to test the ability of FBG to predict the progression of NCL. A *P* value of less than 0.05 was considered statistically significant.

All patients were divided into the control group (without NCL progression) and the progression group (with NCL progression) in accordance with the definition of NCL progression.

## Results

**Fig. 1:**
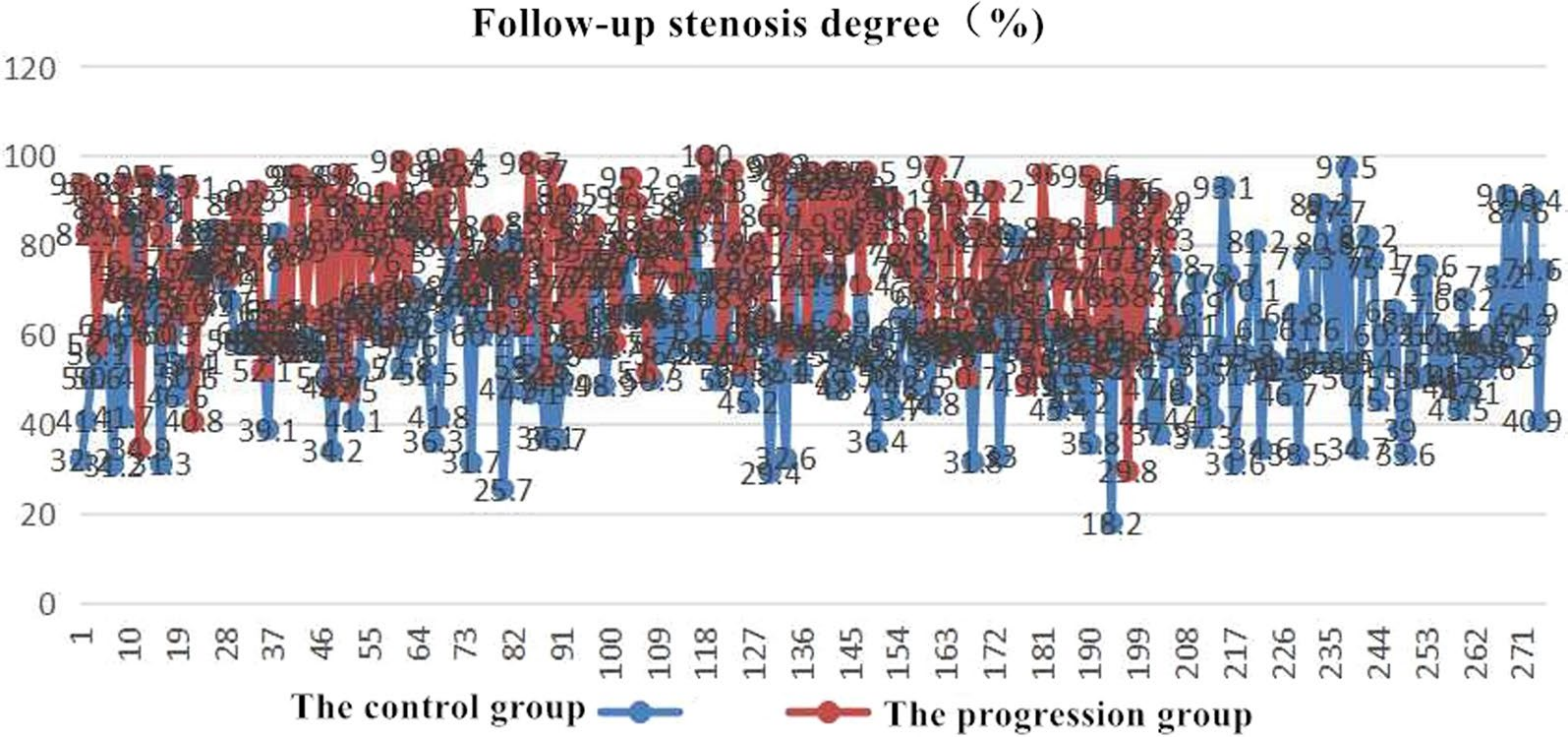
Follow-up quantitative coronary angiography

There were 205 (177 men and 28 women) patients without NCL progression (the control group) and 275 (222 men and 53 women) patients with NCL progression (the progression group) (Fig. 1).

There were no significant differences in age, sex, history of diabetes mellitus, and rates of hyperlipidemia, smoking, myocardial infarction, PCI, CABG, heart rate, systolic arterial pressure, left ventricular ejection fraction (LVEF), cardiac troponin I (cTnI) peak value, triglyceride (TG), total cholesterol (TCHO), high density lipoprotein cholesterol (HDL-C), low-density lipoprotein cholesterol (LDL-C), total three-triiodothyronine (TT3), total thyroxine (TT4), free triiodothyronine (FT3), C reactive protein (CRP), uric acid (UA), time from attack to reperfusion, myocardial blush grade (MBG) of 0–1 in the culprit artery, pre-dilation rate, thrombotic lesion rate, ≥ two vessel lesion rate, collateral circulation rate, culprit lesion length, complex lesion rate, and degree of baseline stenosis between the two groups (all *P* > 0.05) (Table 1).

The average of LVEF seem to be higher than expected ( 53% in average, in both groups) for STEMI patients. It may be involved in the Teichholz method.

There were significant differences in BMI (*P* < 0.001), FBG (*P* < 0.001), glycated albumin (GA) (*P* < 0.01), hemoglobinA1c (HbA1c) (*P* < 0.01), homocysteine (Hcy) (*P* < 0.001), serum creatinine (Scr) (*P* < 0.001), and the degree of follow-up stenosis(*P* < 0.001) between the two groups (Table 1).

In terms of medications, patients received a similar amount of β-blockers (62% vs. 65%), calcium antagonists (30% vs. 28%), ACEI/ARB (56% vs. 54%), and statins (91% vs. 89%) in each group (all *P >* 0.05) (Table 1).

Multivariate logistic regression analysis indicated that FBG (OR 1.274, 95% CI 1.077–1.505, *P* = 0.005) and Scr (OR 1.020, 95% CI 1.002–1.038, *P* = 0.027) were independent predictors of the progress of NCL after primary PCI in patients with STEMI (*P* < 0.05) (Table 2).

Partial correlation analysis showed that FBG was positively correlated with NCL progression (*r* = 0.231, *P* = 0.001) .

**Fig. 2:**
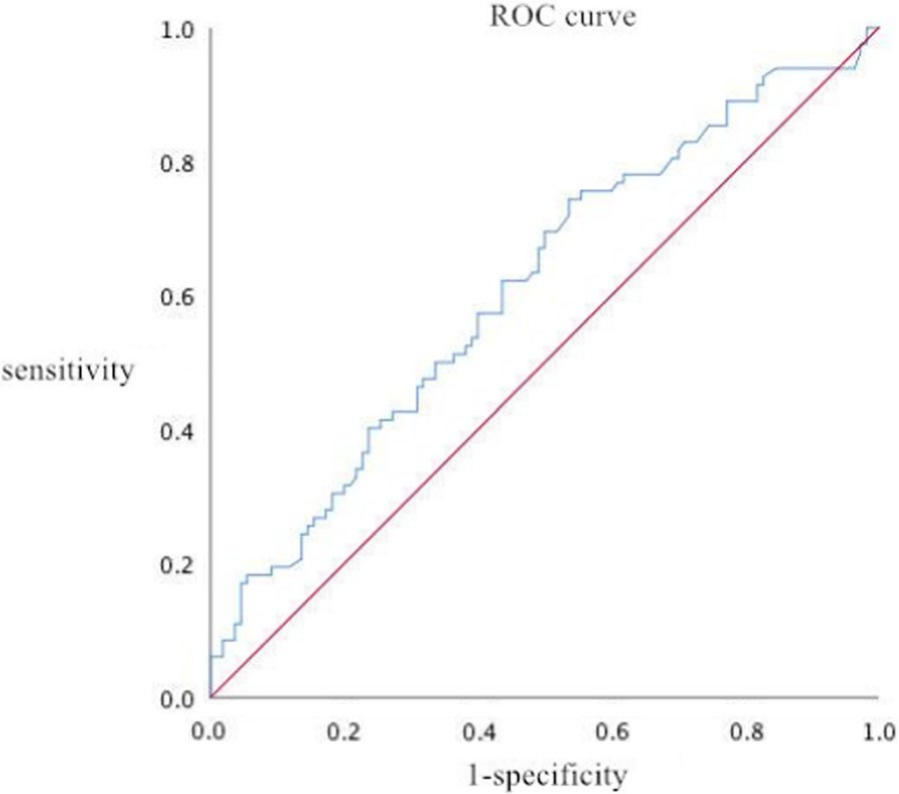
ROC curve for the predictors of NCL progression

ROC analysis for the predictors of NCL progression indicated that an FBG level ≥ 5.715 mmol/L may predict NCL progression. The sensitivity was 74.4% and the specificity was 76.6% (*AUC*: 0.613, SEM: 0.041, 95% CI 0.532–0.693, *P* < 0.008) (Fig. 2).

**Table 1:** Baseline clinical and angiographic characteristics

	The control group (*n* = 275)	The progression group (*n* = 205)	*P* value
Age (y) Male (%) BMI (kg/m^2^) Diabetes mellius (%) FBG (mmol/L) GA (%) HbAlc/% Hypertension (%) Hyperlipidemia (%) Current smoking (%) Prior myocardium infarction (%) Prior PCI (%) Prior CABG (%) β-blockers (%) Calcium antagonists (%) ACEI/ARB (%) Statins (%) Heart rate, beats/min Systolic arterial pressure(mmHg) LVEF (%) cTnI peak value (ng/ml) TG (mmol/L) TCHO (mmol/L) HDL-C (mmol/L) LDL-C (mmol/L) Hcy (umol/L) TT3 (nmol/L) TT4 (nmol/L) FT3 (pmol/L) CRP (mg/L) Scr (umol/L) UA (umol/L) Time from attack to reperfusion (min) MBG 0–1 Predilation rate Thrombolic lesion rate (%) ≥ 2 vessle lesion rate (%) Collateral circulation rate (%) Culprit lesion length (mm) Complex lesion rate (%) Baseline stenosis degree (%) Follow-up stenosis degree (%)	59 ± 9 222 (80.7%) 25.5 ± 2.9 70 (25.5%) 6.4 ± 1.6 15 ± 3 6.3 ± 1.4 150 (54.5%) 75 (27.3%) 177 (64.4%) 15 (5.5%) 25 (9.1%) 0 170 (62%) 82 (30%) 152 (55%) 250 (91%) 82 ± 13 133 ± 23 53 ± 11 21.76 ± 3.55 1.9 ± 1.3 4.3 ± 1.1 1.00 ± 0.23 2.6 ± 0.9 15 ± 8 1.39 ± 0.34 109 ± 24 4.9 ± 0.7 3.36 ± 1.01 71 ± 16 346 ± 90 371 ± 172 113 (41.10%) 85 (30.91%) 68 (24.73%) 130 (47.27%) 70 (25.45%) 29.3 ± 13.1 110 (40.00%) 32.1 ± 13.1(bb) 60.2 ± 14.3(B)	58 ± 10 177 (86.3%) 26.7 ± 3.6 57 (27.8%) 7.2 ± 2.2 16 ± 4 6.7 ± 1.3 130 (63.4%) 57 (27.8%) 130 (63.4%) 20 (9.8%) 25 (12.2%) 0 132 (64%) 58 (28%) 110 (54%) 183 (89%) 83 ± 12 131 ± 21 53 ± 10 22.12 ± 3.61 2.0 ± 1.2 4.3 ± 1.1 0.98 ± 0.20 2.6 ± 0.9 18 ± 11 1.38 ± 0.26 109 ± 16 4.8 ± 0.6 3.55 ± 1.93 77 ± 21 348 ± 97 373 ± 178 85 (41.46%) 67 (32.68%) 52 (25.37%) 102 (49.76%) 52 (25.37%) 29.7 ± 13.5 80 (39.02%) 34.4 ± 13.6(aa/a) 78.3 ± 15.4(A)	0.259 0.104 < 0.001 0.564 < 0.001 < 0.01 < 0.01 0.051 0.897 0.830 0.073 0.271 – 0.564 0.716 0.725 0.550 0.390 0.329 1.000 0.276 0.390 1.000 0.320 1.000 < 0.001 0.725 1.000 0.101 0.163 < 0.001 0.816 0.901 0.935 0.678 0.873 0.590 0.982 0.744 0.829 0.062 < 0.001

Data are presented as n (%) or mean ± SD unless other indicated. BMI: body mass index; FBG: fasting blood glucose; GA: glycated albumin; HbA1c: hemoglobin A1c; PCI: percutaneous coronary intervention; CABG: coronary artery bypass grafting; ACEI/ARB: angiotensin-converting enzyme inhibitor/angiotensin II receptor blocker; LVEF: left ventricular ejection; TG: triglyceride; TCHO: total cholesterol; HDL-C: High density lipoprotein cholesterol; LDL-C: low-density lipoprotein cholesterol; Hcy: homocysteine; Scr: serum creatinine; TT3: total three-triiodothyronine; TT4: total thyroxine; FT3: free triiodothyronine; CRP: C-reactive protein; Scr: creatinine; UA: uric acid; MBG: myocardial blush grade

**Table 2:** Multivariate logistic regression analysis

Factor	*B* value	*SE* value	*OR* value	95% CI	*P* value
Scr FBG BMI	0.20 0.242 0.089	0.009 0.085 0.049	1.020 1.274 1.093	1.002–1.03 1.077–1.50 0.992–1.203	0.027 0.005 0.071

OR: odds ratio; CI: confidence interval; Scr: serum creatinine; FBG: fasting blood glucose; BMI: body mass index

## Discussion

PPCI in a culprit artery is the preferred strategy for treating patients with acute STEMI. However, approximately 40–65% of patients with STEMI present with three-vessel lesions. A clinical follow-up study of patients with three-vessel lesions after successful PCI suggested that NCL progression may be occurring [1]. This may be the most important factor that affects the prognosis of patients with acute myocardial infarction after successful PCI.

However, there have been few studies on the clinical predictors for progression of NCLs. Tsiamis et al. [6] performed follow-up angiography in 117 patients with acute coronary syndrome. These authors suggested that NCLs may have progressed, and acute myocardial infarction may be an independent predictive factor for the progression of NCLs.

Our follow-up study on the progression of NCLs suggested that this progression may be the most important prognostic factor in patients with STEMI after successful PCI [7]. Our data suggested that the progression of NCLs could involve inflammation and a stress mechanism, and a high dosage of ramipril may inhibit NCL progression, which could be the main cause of revascularization after PPCI for patients with STEMI.

In the present study, we investigated the clinical prediction factors of NCL progression in patients with STEMI after PPCI. We carried out a 12-month clinical and angiographic follow-up in 480 patients, and found that there were significant differences in BMI, FBG, GA, HbA1c, Hcy, and Scr between the control group and the progression group, indicating that BMI, FBG, GA, HbA1c, Hcy,and Scr may be clinical correlation factors of NCL progression in patients with STEMI after PPCI.

Elevated plasma Hcy is an independent risk factor for atherosclerosis, which is recognized as inflammatory and immune responses. In the present study, we suggest that Hcy promote NCL progression by regulating inflammatory and immune responses.

In the current study, there were no significant differences in the patient characteristics and medical history between the control group and the progression group. Additionally, all patients received comparable medication.

Multivariate logistic regression analysis indicated that FBG and Scr were independent predictors of the progress of NCL after primary PCI in patients with STEMI. It showed glycometabolism disorder and renal insufficiency may promote the progress of NCL after primary PCI in patients with STEMI.

Partial correlation analysis showed that FBG levels were positively correlated with NCL progression. ROC analysis for the predictors of NCL progression indicated that a fasting glucose level ≥ 5.715 mmol/L may predict NCL progression, and the sensitivity was 74.4% and the specificity was 76.6%. These results indicated that elevated FBG may be an independent predictor of NCL progression in STEMI patients who underwent primary PCI.

Both the R square value of the partial correlation analysis and the AUC of the ROC curve were rather low, in particular the AUC which is just over 60% and whose lower limit is just 0.532,

although there were significant difference. This may be related to patients that enrolled were not enough in this study.

- The cut-off for blood glucose, which also involves satisfactory sensitivity and specificity, is 5.715 mmol/L, and very close to normal values. It indicated that strict glycaemic control is needed in the post-infarction period, and consequently an aggressive treatment to keep glycaemia low is recommended.

Diabetes is an independent risk factor for coronary artery disease. Compared with the non-diabetic population, diabetes is associated with a two–three-fold increase in the risk of cardiovascular disease and mortality because of cardiovascular disease [6]. Previous studies have shown that glucose metabolism plays a role in the development and prognosis of coronary heart disease. Even patients with mildly elevated blood glucose levels are more prone to acute myocardial infarction than patients with healthy blood glucose levels [7]. Berry et al. [8] found that FBG, HbA1c and a history of diabetes are associated with the severity and progression of coronary atherosclerosis. In the current study, fasting glucose and creatinine levels were found to be independent predicters of NCL progression, and fasting glucose was positively correlated with NCL progression in a partial correlation analysis. Increased secretion of high levels of catecholamine, glucocorticoids and other hormones in acute myocardial infarction can enhance liver glycogen decomposition and inhibit glycogen production [9].In addition to upregulating glucose production, insulin resistance and impaired glucose uptake mechanisms during critical disease jointly lead to the occurrence of hyperglycemia [10, 11]. Hyperglycemia leading to plaque progression may involve the following mechanisms [12] : (1) Non-enzymatic glycation of proteins and lipids increases, and the formation of reactive higher glycation end products, resulting in mechanical dysfunction of the vascular wall. This obstructs circulating blood cells and causes them to adhere to the blood vessel wall, and also interferes with cell function by binding to a variety of receptors on macrophages, endothelial cells and other cells, increasing pro-inflammatory signal transduction and promoting inflammation of the blood vessel wall. (2) During hyperglycemia, insulin receptor substrate 1 is down-regulated and the cells become resistant to insulin. The insulin-like growth factor 1 receptor sends signals through other alternative scaffold proteins to induce vascular smooth muscle cells to dedifferentiate, migrate and proliferate. (3) By activating protein kinase C, hyperglycemia causes many abnormal changes related to atherosclerosis, such as increased vascular permeability, endothelial dysfunction and reduced production of nitric oxide, resulting in impaired vasodilation, increased apoptosis, and increased production of extracellular matrix.

## **Conclusion**

The results of the current study suggest that BMI, FBG, GA, HbA1c, and Hcy may be clinical correlation factors for NCL progression in patients with STEMI after PPCI. FBG and Scr were independent predictors of the progress of NCL after primary PCI in patients with STEMI. This study is a single-center retrospective analysis, with a relatively small sample size and a lack of detailed intravascular imaging data, and there is a need for further randomized prospective controlled studies.

## Data Availability

Please contact author for data requests.

## Abbreviations

STEMI: ST-segment elevation myocardial infarction
PPCI: Primary percutaneous coronary intervention
BMI: Body mass index
FBG: Fasting blood glucose
GA: Glycated albumin
HbA1c: Hemoglobin A1c
PCI: Percutaneous coronary intervention
CABG: Coronary artery bypass grafting
ACEI/ARB: Angiotensin-converting enzyme inhibitor/angiotensin II receptor blocker
LVEF: Left ventricular ejection
TG: Triglyceride
TCHO: Total cholesterol
HDL-C: High density lipoprotein cholesterol
LDL-C: Low-density lipoprotein cholesterol
Hcy: Homocysteine
Scr: Serum creatinine
TT3: Total three-triiodothyronine
TT4: Total thyroxine
FT3: Free triiodothyronine
CRP: C-reactive protein
UA: Uric acid
MBG: Myocardial blush grade
